# Prospects for the Use of Social Media Marketing Instruments in Health Promotion by Polish Marshal Offices

**DOI:** 10.3389/fpubh.2018.00065

**Published:** 2018-03-07

**Authors:** Magdalena Syrkiewicz-S´witała, Piotr Romaniuk, Agnieszka Strzelecka, Katarzyna Lar, Tomasz Holecki

**Affiliations:** ^1^Department of Health Economics and Health Management, School of Public Health in Bytom, Medical University of Silesia in Katowice, Bytom, Poland; ^2^Department of Health Policy, School of Public Health in Bytom, Medical University of Silesia in Katowice, Poland, Bytom, Poland; ^3^Faculty of Medicine and Health Sciences, Institute of Public Health, Jan Kochanowski University in Kielce, Kielce, Poland

**Keywords:** marketing communication, social media, social media marketing, health promotion, public health

## Abstract

**Purpose:**

To investigate whether the Polish Marshal Offices use instruments for social media marketing activities in the field of health promotion.

**Methodology:**

14 Polish Marshal Offices participated. The Computer-Assisted Web Interview and Computer-Assisted Telephone Interview were used along with a proprietary questionnaire. Standard statistical methods were employed.

**Findings:**

The number of people using the Internet and social media in Poland is steadily growing. The majority of the offices (93%) performed health promotion activities. The authorities collaborated with other units of local government and non-governmental organizations in these activities. According to respondents, the most convincing form of health promotion is direct communication (46%). More than half of the surveyed offices (56%) did not use portals or social networking sites in health campaigns. The rest of the offices indicated using Facebook (25%) or YouTube (6%). Half of them did not apply the tools of social media marketing. The other half was involved in discussions on health-related online forums (moderation or consulting). Relatively few offices use social media and social media marketing in health promotion campaigns.

**Value:**

The use of social media by the Marshal Offices may result in a potential increase in effectiveness of the pro-health campaigns. It is recommended that Polish Marshal Offices recognize the potential benefits of social media marketing campaign instruments in the field of health promotion in order to reach out the digital recipients.

## Introduction

Health promotion is “the process of enabling every person to increase the impact on his/her health in the sense of its improvement and maintenance” (1). These activities are designed to provide access to knowledge that promotes health-related behavior. Then, this knowledge creates the conditions necessary to take appropriate decisions to improve population-wide health. All these activities should strengthen the process of increasing social responsibility for the health of the population. It is important to involve local, regional, and state institutions in improving these conditions. Their duty is to face societal expectations in the area of health and to influence and impact the community to participate actively in individual health-related activities (2). To achieve these objectives, they can use the most common and current communication tools and channels of communication. Today, the Internet and social media have become a common medium, allowing for flow of all types of information, including health data. Social media, generated by its users, change the way of communication and spreading information. These media are characterized by self-sufficiency and systematically increasing number of recipients (3). The development and prevalence of electronic mass media and the dynamic progress of mobile communication affect how individual units of local communities and state institutions function (4). Social media creates an efficient way to communicate with target groups for promotion and health education (5). It allows for quick responses to undesirable social phenomena and testing of new approaches in this field (6). The validity of this message is reliability of the information. The main allegation against the use of this media in important social issues such as crisis situations or the dissemination of health information was lack of credibility. This is due to the inability of media users to control the content being passed on. It is currently determined that there is the possibility of crediting the transmitted content (7). It is necessary for users to use models that assist in discovering reliable information. It is also believed that this may be achieved automatically by features extracted from information cascades (7). In the era of digitization, using social media among state institutions in carrying out their duties and responsibilities in the field of promotion and health education seems to be an effective strategy. Thus, it is necessary to establish whether Marshals’ Offices use social media tools and social media marketing in communication for health promotion.

### Responsibilities and Tasks of the Polish Marshal Offices in the Field of Health

The Polish Marshal Offices are units of regional public administration, whose task is to implement, in cooperation with various partners, laws and their own tasks aimed at development of a specific province. Self-governed provinces are responsible for the implementation of activities in the area of health according to the law (8), and they are responsible for the development, implementation, and evaluation of the effects of health policy programs, after consultation with the relevant territorial municipalities and counties (9). In addition, at the discretion of self-governed provinces, tasks for the prevention of alcohol problems (10) and protection against the consequences of tobacco use belong under the provision of the applicable laws (11).

The Marshal Offices are auxiliary apparatuses for the management of the province, and they are obliged to create appropriate conditions for carrying out provincial tasks set out in their strategy. Health care falls under these tasks, including the following elements (12):

– analyzing data concerning health and epidemiological, demographic, and economic factors and the availability and quality of services implemented in the region by health-care establishments;
– coordination and control issues related to the functioning of care and treatment and nursing care;
– planning, initiating, and coordinating activities in the field of health promotion, health education, and the prevention and control of diseases of social importance;
– fulfillment and realization of the tasks of prevention and solving problems of substance abuse and violence prevention in line with the needs of inhabitants of the province;
– creating and coordinating the computerization of subordinate units (e.g., offices of municipalities, counties, provincial) in terms of interoperability.

These units, performing tasks in the area of health, seek to adjust to the changing conditions of the public administration and its relationship with the environment. Their main objectives include health promotion actions, disease prevention, effectiveness of implementation of the tasks assigned and, above all, wide access to the target groups. Therefore, the current Marshal Offices are trying to computerize the process of working in this field in order to better adapt to the expectations of contemporary audiences. In the face of the continuous process of improving population health, it has become important for Marshal Offices to use all available methods and instruments conducive to achieving the desired objectives, including the Internet. *Via* the Internet and social media, and using the tools of social media marketing, the sender can monitor the behavior of recipients and track opinions.

### Social Media to Promote Health

The Internet is an efficient and effective channel of communication that allows two-way communication, allowing for content exchange among its users (13). Internet users focus their activity on networks that represent the concerns of their subjects or purchased goods (products or services). People use social media for self-education and information search (14). They seek information applicable to issues of health in general, and build a network of relationships by creating social media focused on a particular health problem. Thus, they form groups with similar problems or interests. Each participant of the group co-creates the published content and has the opportunity to share his/her own opinion or experience. The increase in interest in this mode of communication is also supported by an expanded range of mobile devices, which allow for faster spread of information and reduced cost of communications (15). The development of social media affects the development of Internet marketing tools, and in particular, social media marketing (16). Social media marketing is “a set of relationships, behaviors, feelings, empiricism, and the interaction between consumers, brands, where the omnidirectional communication to exchange experiences with advanced communication tools” (17). The importance of social media is becoming increasingly important in mobile technology. Social media provides not only entertainment but also support for the decision-making process while purchasing certain goods and ideas. Companies, enterprises, and institutions use these trends to direct their social media marketing strategies (18). These media are useful source of information, but it is also difficult to understand properly and interpretively in the context of determining the attitudes or motives of recipients of the content (19). The marketing efforts conducted in this medium require other means of communication to achieve the intended goal. The aim is not only to build relationships, good image, or strengthen business messages but also to create new beliefs and attitudes (20). Social media thus becomes a space for activity in the field of promotion and health education.

### Study Objectives

The main objective of the study was to identify health promotion activities of the Marshal Offices and ways to implement the tasks undertaken with the use of Internet marketing tools, and in particular, in social media marketing. The detailed purposes are as follows:

– to define fields of action of Polish Marshal Offices in the area of health promotion;
– to determine the benefits and difficulties arising from cooperation with non-government organizations (NGOs) and local governments;
– to select and identify marketing communications tools, including social media marketing, used by the Polish Marshal Offices in projects in the field of health promotion;
– to develop recommendations to improve the effectiveness of communication by Polish Marshal Offices in the field of health promotion.

## Materials and Methods

A nationwide survey was sent to all 16 Polish Marshal Offices. The research was conducted in 14 offices that agreed to participate. Purposeful sampling was used. Respondents were matched in terms of knowledge, competence, and experience relevant to the subject of health. Closed questions related to the implementation of health promotion activities, and open-ended questions focusing on their cooperation and effective communication in health-related tasks with other authorities (provincial, municipalities, and counties) and NGOs.

The research material was collected using online survey techniques (Computer-Assisted Web Interview) and telephone interviews (Computer-Assisted Telephone Interview). Data collection followed several stages. Invitations to participate were sent electronically. The call consisted of a cover letter, information about the project implemented, and hyperlinks to the questionnaire. For greater maneuverability, completed questionnaires were sent in three mailings at specified intervals and then sent to the studio call center. The study did not apply advanced analytic methods in connection with the baseline performance analysis on qualitative variables.

Advanced calculation methods were not applied in this article. It is caused by usage of qualitative variables. To improve the accuracy of the tests, a statistical analysis was conducted with the use of the Pearson’s chi-square test with the Yates correction. In this article, Pearson’s chi-square test evaluated the significance of differences between the respondents’ answers from two groups: Marshal Offices operating in the eastern Poland and in the western Poland. This division was made because of the economic differences of the Polish regions. The result of statistical test was the test probability (p), which low values may indicate statistical significance of considered differences. The level of statistical significance was set at *p* < 0.05.

## Results

Most of the responding Polish Marshal Offices (13; 92.9%) declared commitment to health promotion. The Marshal Offices must work with other stakeholders in actions to protect health. The main partners were other local government units (counties and municipalities) and NGOs. Respondents were asked to indicate the main areas of mutual cooperation and its advantages and disadvantages. The areas of mutual cooperation in health mainly focused on cancer prevention as well as promotion and health education. The activities of health care (i.e., disease prevention, promotion, and health education in the perception of the respondents) were often referred to as potential benefits resulting from cooperation with both districts and municipalities, as well as with NGOs. Most local government (*N* = 11; 78.6%) saw potential in possible cooperation with other entities. In their mutual relations, a lack of obstacles to communication was emphasized (*N* = 8; 57.1% of municipalities and counties; *N* = 7; 50% of NGOs). However, they drew attention to the difficulties arising from the complicated legislation, particularly with regard to NGOs (*N* = 9; 64.3%), as well as excessive bureaucracy and complicated and complex procedures for all potential partner relationships (*N* = 11; 78.6% NGOs, *N* = 9; 64.3% municipalities, *N* = 7; 50% counties). Most Marshal Offices (*N* = 12; 85.7%) thought that NGOs could actually relieve offices in performing health promotion tasks, particularly because each of the offices (*N* = 14, 100%) also provides non-financial support to third sector organizations in health care.

Almost all the offices were involved in activities in the field of health promotion (*N* = 11; 78.6%). Special consideration was given to becoming involved in the organization of health promotion campaigns for cancer, primarily cervical cancer and prostate cancer (*N* = 14; 100%) as well as drugs (*N* = 13; 92.9%), with particular concern for alcohol abuse prevention, drugs, and anti-smoking. Next, they focused on prevention of home violence (*N* = 7; 50%) and preventive measures for circulatory system diseases (*N* = 5; 35.7%) and lung disease (*N* = 4; 28.6%). Offices were least likely to take action against obesity among adults (*N* = 2; 14.3%) and children (*N* = 1; 7.1%).

In terms of the most attractive forms of health promotion among patients in the opinion of Polish Marshal Offices, responds could provide more than one answer (results do not add up to 100%). They considered the most attractive and convincing form of health promotion to be direct promotion through friend recommendations (*N* = 12; 85.7%). Another attractive form was considered to be the recommendation of a doctor or pharmacist (*N* = 7; 50%), followed by information posters placed in medical institutions (*N* = 6; 42.9%) and TV spots (*N* = 5; 35.7%). Of less importance were newsletters (*N* = 3; 21.4%) and leaflets (*N* = 3; 21.4%). According to respondents, direct promotion recommendations from other Internet users are also less effective (*N* = 3; 21.4%). The least attractive activities in the area of health promotion respondents indicated: billboards, press releases, radio spots, and Internet adds. Among the tools most frequently used by the Marshal Offices in health promotion campaigns were conferences (*N* = 11; 26.8%), workshops (*N* = 8; 19.5%), initiation of TV programs (*N* = 7; 18%), and the development of information brochures (*N* = 6; 14.6%). The investigated offices were reluctant to use instruments such as the organization of concerts (*N* = 4; 9.8%), development of information leaflets (*N* = 3; 7.3%), and the initiation of radio programs (*N* = 2; 4.9%). Again, respondents could give more than one answer.

**Figure 1:**
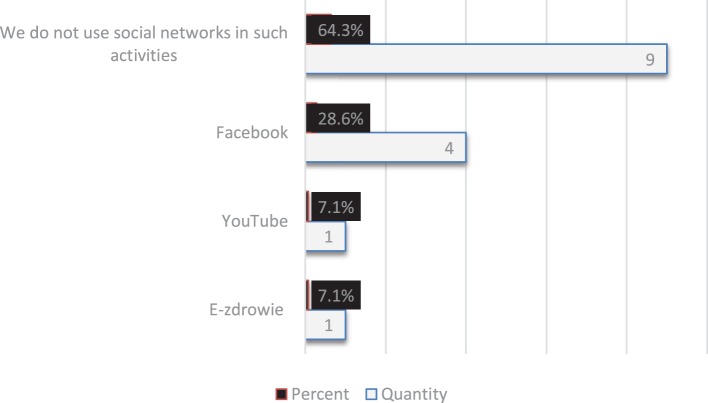
The use of social networks by Polish Marshal Offices in health promotion campaigns (respondents could give more than one answer.)

The tested offices mostly do not use social media, and thus do not use social media marketing campaigns for health promotion. As many as nine of the surveyed institutions (64%) did not use social media. The remaining 5 (35.7%) are seeking benefits from the most popular websites: Facebook, YouTube, and E-zdrowie (en. E-health) (Figure 1).

**Figure 2:**
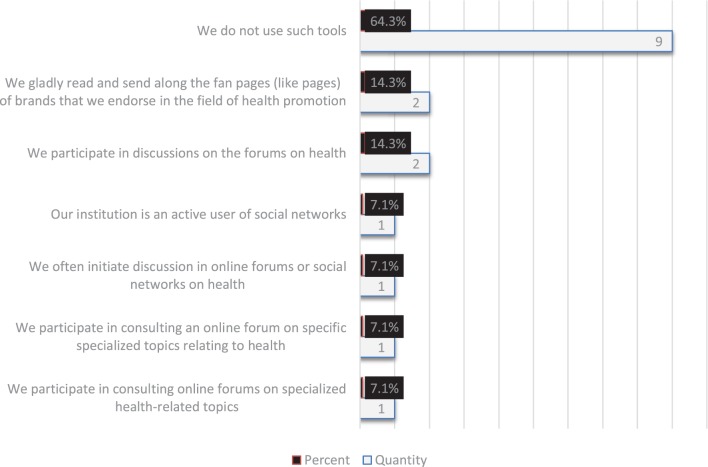
Use of social media marketing tools by Polish Marshal Offices in health promotion campaigns (respondents could give more than one answer.)

The social media marketing tools chosen by Polish Marshal Offices were analyzed in detail. Only two offices (14.3%) participated in discussions on online forums about health and were eager to read and forward the fan page (like page) with recommended brands for promoting health. Offices grant individual declaration (7.1%) in moderating online health forums, frequently initiate discussions on online forums or social networks on health, or consult online forums on specialized health topics. Only one institution (7.1%) was an active and committed user of social networking sites (Figure 2).

No statistically significant differences were showed between Marshal Offices functioning in the East and the West of Poland in the area of the analyzed issue, i.e., in the scope of mutual cooperation with other offices of state administration or non-governmental organizations, as well as between the choice of tools for health promotion or the frequency and method of usage the tools of social media marketing and distribution of healthy information through them. In none of these issues, results of statistical analyzes reached low values (below *p* < 0.05), which would demonstrate a statistical significance of considered differences.

## Discussion

Only half of the respondent office use social media to communicate with their key stakeholders: citizens, other offices of public administration, and non-governmental organizations.

The vast majority of Polish Marshal Offices are involved in health promotion activities. Cooperation with other entities may be implemented primarily in the field of health promotion and disease prevention. Not all marshal offices use in these relations social media. The value of cooperation with other local governments at lower levels and NGOs pointed to community involvement, knowledge of health needs, and access to a wide audience. The perceived benefits of cooperation included building contacts with qualified staff and relief worker offices. Such cooperation with local government units, however, may have led to excessive bureaucracy and complicated procedure. The survey carried out in 10 selected countries (Brazil, Germany, India, Norway, Singapore, South Korea, the Kingdom of Saudi Arabia, the United Arab Emirates, United Kingdom, and United States) shows that social media and mobile digital technology greatly reduce the bureaucratic problems in public administration. They improve information flow and workflow significantly that reduces bureaucratic problems (21). There is also estimated data related to 23 European governments, which show that open to new channels of communication web office can reduce administrative costs by up to 15–20% (22). Social media have great potential for use by public administration institutions in relations with specific target groups (23).

Social media allow to disseminate useful content, enforce laws and regulations, and support the process of mass collaboration for different prosocial purposes (24). However, there is one requirement: high level of commitment in social media and knowledge of the cultural aspects (24). There are three stages of commitment (24). The first is to start using social media, consisting of uncoordinated exchange of information. The second is the creation of platforms for the exchange of information, organization of communication in social media. The third is advanced and mature. It is to utilize social media for different purposes (24). Polish Marshal Offices are at the initial stage of use of social media.

In the area of health promotion, respondents focused on cancer and stimulants. The majority frequently used conferences and workshops as communication tools. According to half of the respondents, the most convincing form of patient health promotion is direct communication, especially recommendations from friends, and less important, information posters and TV spots. Despite this awareness, more than half of the surveyed offices did not use portals or social networking sites for health campaigns. About one quarter of the offices used Facebook, the most popular social networking site. The rest did not use any social media marketing tools. The other half tried to engage in discussions and moderation of online forums and consultation in the field of health. This study shows that the Polish Marshal Offices do not follow modern trends in communication, in terms of actions for health. The tested offices showed insufficient use of social media tools and marketing. Less than half of the surveyed offices used it in their efforts to promote health.

As other studies prove, the present way of communication between public administration and citizens should be expected to change. It should be more transparent and open for modern trends of communication (25). This process should be supported by the activity of offices on the Internet also through participation in social media (26). The openness of the authorities to the citizens is a significant problem of choosing the channels of communication, its future is unknown, but predictable. The governments of Denmark and the UK already recognize the importance of digital interaction in relations with citizens (27). It is observed that although social media do not dominate completely the form of personal or telephone contact, they support the process of solving current official affairs (27, 28). It is supposed that the digital involvement of citizens will translate well to the area of health. Nowadays, social media is a place to search for information on health. Currently, it is estimated that, e.g., Facebook is one of the more common sources of health information for residents of USA or the UK. It is determined that it is on a fourth position concerning popularity (29). Polish Internet users seek content on health as well (30). It should be noted that 67% of Poles use the Internet, 36% of them use social media (31), and 88% are looking for online health information (32), and these numbers are steadily increasing.

Unfortunately, the Polish Marshal Offices are insufficiently present in the net. Tested offices, in the era of ubiquitous Internet, lack significant use of social media tools and social media marketing. Less than half of the surveyed offices actually use it in their efforts to promote health and communication with all its stakeholders (not just citizens). Other researches show that the digital modes of communication, including social media, can deliver real benefits for society and the economy, also with regard to all sorts of public services (21). The digital modes of communication can also support the dialog between the authorities and the stakeholders, helping to learn real expectations and needs (33). They drive to a satisfactory and positive interaction in various fields of activities in the public sector (23, 34).

## Conclusion

The main fields of activity of tested Marshall Offices in the area of health promotion are cancer and drugs. Polish Marshal Offices do not fully utilize digital media or in general do not use them. Few offices use social networking site Facebook in health promotion campaigns or try to participate in discussions on online forums or moderate them. As advantages of cooperation with other entities (offices or NGOs) in the field of health shall specify: knowledge of the needs of local community and better access to the target group. The main disadvantages in cooperation are bureaucracy and non-transparent procedures.

It is recommended that the Polish Marshal Offices recognize the potential of social media tools and social media marketing campaigns for health promotion. The dynamic development of digital technologies should affect their functioning in terms of actions to protect health. As a result, authorities will be able to capitalize on the many areas of its operation within modern communication technology, provided that they endorse modern communication trends. Currently, it is necessary to reach out to the digital audience with pro-health messages and thereby fulfill statutorily imposed duties and responsibilities in the field of health. It is postulated, therefore, that offices were not closed off from the use of modern communication techniques, and this did not lead to “digital divide.” The Polish Marshal Offices cannot allow a situation where developed health promotion campaigns become archaic and are no longer noticed by potential receivers, and thus be contrary to the expectations of people active on the Internet and using social media.

It is highly recommended that Marshal Offices in Poland develop their ability to use actively social media marketing in conducted projects. Since a specific knowledge and experience is required, the Marshall Offices may consider hiring a professional social media company. The future social media tools and campaigns might be supported by spreading the knowledge on performed actions among doctors, pharmacists, and other public persons. It is expected that the number of people active in social media will grow steadily. It is high time for Marshall Offices to start active presence in the Internet because the target groups are there already.

## Author Contributions

MS-Ś conceived the study and prepared draft of the paper. MS-Ś, PR, and AS contributed to paper preparation and study. KL and TH provided new information necessary to revise the paper.

## Conflict of Interest Statement

The authors declare that the research was conducted in the absence of any commercial or financial relationships that could be construed as a potential conflict of interest.
